# Numerical and Experimental Investigations of Fracture Behaviour of Welded Joints with Multiple Defects

**DOI:** 10.3390/ma14174832

**Published:** 2021-08-25

**Authors:** Mihajlo Aranđelović, Simon Sedmak, Radomir Jovičić, Srđa Perković, Zijah Burzić, Dorin Radu, Zoran Radaković

**Affiliations:** 1 Innovation Centre of the Faculty of Mechanical Engineering, 11120 Belgrade, Serbia; Mixaylo23@gmail.com (M.A.); simon.sedmak@yahoo.com (S.S.); rjovicic@mas.bg.ac.rs (R.J.); 2 Military Technical Institute, 11030 Belgrade, Serbia; perkovic.srdja@gmail.com (S.P.); zijah.burzic@vti.vs.rs (Z.B.); 3 Faculty of Civil Engineering, University of Transylvania, 500036 Brașov, Romania; 4 Faculty of Mechanical Engineering, University of Belgrade, 11120 Belgrade, Serbia; zradakovic@mas.bg.ac.rs

**Keywords:** welded joint, finite element method (FEM), multiple defects, stress concentration

## Abstract

Current standards related to welded joint defects (EN ISO 5817) only consider individual cases (i.e., single defect in a welded joint). The question remains about the behaviour of a welded joint in the simultaneous presence of several different types of defects, so-called multiple defects, which is the topic of this research. The main focus is on defects most commonly encountered in practice, such as linear misalignments, undercuts, incomplete root penetration, and excess weld metal. The welding procedure used in this case was metal active gas welding, a common technique when it comes to welding low-alloy low-carbon steels, including those used for pressure equipment. Different combinations of these defects were deliberately made in welded plates and tested in a standard way on a tensile machine, along with numerical simulations using the finite element method (FEM), based on real geometries. The goal was to predict the behaviour in terms of stress concentrations caused by geometry and affected by multiple defects and material heterogeneity. Numerical and experimental results were in good agreement, but only after some modifications of numerical models. The obtained stress values in the models ranged from noticeably lower than the yield stress of the used materials to slightly higher than it, suggesting that some defect combinations resulted in plastic strain, whereas other models remained in the elastic area. The stress–strain diagram obtained for the first group (misalignment, undercut, and excess root penetration) shows significantly less plasticity. Its yield stress is very close to its ultimate tensile strength, which in turn is noticeably lower compared with the other three groups. This suggests that welded joints with misalignment and incomplete root penetration are indeed the weakest of the four groups either due to the combination of the present defects or perhaps because of an additional unseen internal defect. From the other three diagrams, it can be concluded that the test specimens show very similar behaviour with nearly identical ultimate tensile strengths and considerable plasticity. The diagrams shows the most prominent yielding, with an easily distinguishable difference between the elastic and plastic regions. The diagrams are the most similar, having the same strain of around 9% and with a less obvious yield stress limit.

## 1. Introduction

Welded joints are of crucial importance for structural integrity due to their crack sensitivity and material heterogeneity [1,2]. For this reason, welded joints are often locations for stress concentration and crack initiation and growth. The fact that welded joints are typically accompanied by defects (to a lesser or greater extent) further emphasises their importance when assessing the integrity of welded structures. The standards EN ISO 5817 and SRPS EN ISO 6520-1 define the acceptability criteria for welded joint defects, but they consider the presence of a single type of defect in a welded joint. Some other procedures, such as the formerly used PD6493 (which was updated into the BS 7910 standard), consider multiple different defects, but only if they are presented as one large single defect [3]. The subject of multiple defects in welded joints was considered by researchers, but to a far lesser extent. Jovičić et al. [2] pointed out the problems that can occur in welded joints due to their geometry, as defects may cause considerable local increase in stress, possibly resulting in crack initiation. In [1], the same authors analysed the effect of multiple defects on fatigue in the existing standard EN ISO 5817, implying that this widely used standard has additional room for improvement. On the other hand, Kozak et al. [4] considered the influence of stresses induced by linear vertical misalignment of cylindrical parts of a pressure vessel, a case that occurs frequently in practice. Other authors, such as Cerit et al. [5], also focused on the numerical analysis of the influence of defects in welded joints, centred on a specific type of defects—undercuts. As can be seen, even if multiple defects are considered, they are actually repeated single defects, treated using a combination of methods previously mentioned, including both experimental and numerical approaches. Initial steps in this direction can be seen in [6].

In terms of defects, the main focus here is on vertical misalignment of plates, along with excess weld metal, incomplete root penetration, and undercuts. Different combinations of these defects (usually three) are introduced along the length of the welded joint for each plate. The material heterogeneity of welded joints was also taken into account when analysing the behaviour of both experimental test specimens and numerical models made using the finite element method. In addition to the influence of welded joint geometry resulting from the presence of multiple defects on the integrity, the effects of different microstructures in a welded joint should be taken into account, as they can affect the direction in which crack propagation and failure take place. It is of great importance to mention that when analysing the heterogeneity of welded joints, all three zones (the parent material, the heat-affected zone, and the weld metal) should be considered in terms of their different mechanical properties resulting from the welding procedure itself. In this case, only the weld metal and the parent material were taken into account, whereas the heat-affected zone will be observed separately in future experiments and numerical analysis once the approach described here is sufficiently developed and verified.

Hence, it can be seen that the ultimate goal of this paper (and the research that it is a part of as a whole) is to unify all factors that influence the behaviour and integrity of welded joints and structures in the presence of multiple defects, with a particular focus on those that occur frequently in practice.

The research presented in this paper involved a number of stages, which will be explained in detail—starting with the welding of plates with specific defect combinations, followed by numerical simulations based on the dimensions measured on the welded plates, after which the tensile tests were performed, with the goal of verifying the obtained numerical results. Once these comparisons were made, it was concluded that additional analyses are necessary for some numerical models and their corresponding specimens in order to improve the existing models so that they would represent the specimen behaviour more realistically.

Compared with other studies [6], the present research is considering a different approach—taking into account defects and a combination of defects, combining experimental and numerical method approaches, thus assessing the structural integrity of the effect of multiple different defects in the welded joint.

## 2. Materials and Methods

The methods used for these investigations represent a combination of tensile testing of welded joint specimens made of common low-carbon low-alloy steel and numerical simulations of the same specimens. Thus, the main approach was to combine experimental and numerical methods in order to determine and describe the effect of multiple different defects simultaneously present in the welded joint on its structural integrity.

The parent material used in the research is a common structural steel, S235JR steel [6], with the aim of developing the research methodology, which will later be used also for higher-strength steels. Table 1 and Table 2 show the chemical composition and mechanical properties of S235JR steel, respectively. The wire VAC 60 was used as a filler material due to its good mechanical properties (Table 3), suitable chemical composition for the purpose of this investigation (Table 4), and availability. This combination of parent and filler materials resulted in significant overmatching since its yield stress was well above that of the parent material. This fact, along with nonstandard welded joint geometry, largely contributed to the results obtained by both numerical and experimental analyses.

Regarding the preparation of the tensile test specimens, three specimens with a length of 200 mm and a cross section of 25 × 10 mm^2^ were cut out from each group of defects. The welded plates were made of two pieces with 500 × 200 mm dimensions, which were then welded with one defect combination in the first half and a different combination in the second half. As a result, four different defect combinations were obtained for half a welded plate each. Hence, a total of 12 specimens were subjected to tensile testing in order to obtain more accurate results in accordance with relevant standards.

The welding parameters that were used for the MAG procedure are shown in Table 5 and Table 6 for welded joints with and without misalignment. As regards the filler material used, the VAC 60 wire diameter was 1.6 mm.

**Figure 1:**
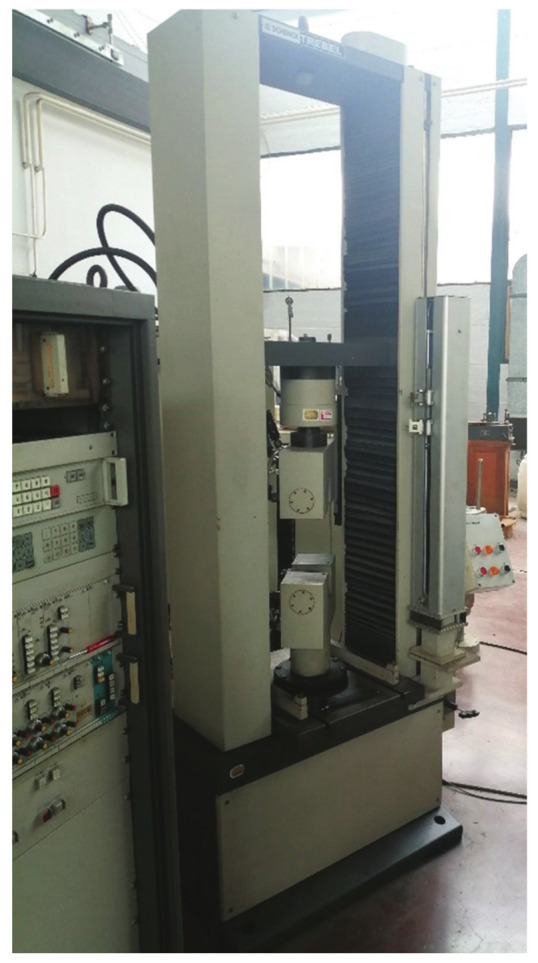
Instron tensile test machine used for the experiments.

The tensile tests in this research were performed at the Military Technical Institute in Belgrade on an Instron tensile test machine (load capacity of 250 kN). The tensile testing equipment that was used are shown in Figure 1. The test specimens were divided into four defect combination groups. As can be seen in the figure, the specimens were not machined, and the original geometry obtained during welding was preserved. The cross-section area of the specimens was 250 mm^2^ (25 × 10), whereas the expected tensile strength was around 360 MPa. Based on these values, it was determined that failure of the specimens should be expected at load levels of 90–95 kN, which was confirmed by an experiment. Tensile tests were thoroughly monitored, including the making of images of all four types of specimens, which will be shown at a later point in this paper. As a result, force-displacement diagrams were obtained, which can then be used to determine the stress–strain curves for the needs of numerical simulations.

Experiment preparations were performed in accordance with the EN ISO 15614-1:2017 standard (welding specification and qualification), and the specimens were also cut according to this standard’s recommendations. With regard to the tensile test experiment, it was performed based on the standard PN-EN ISO 4136:2013 for destructive testing of welded joints in metallic materials, which is closely related to the EN ISO 6892-1:2020 standard, commonly used for such applications.

Finite element methods were used for numerical simulations due to their simplicity, efficiency, and repeatability, as shown in [6,7,8,9,10,11,12,13,14,15,16], where Abaqus 2017 was used in similar analyses. Both elastic and plastic behaviours were defined in these models, for the parent material and weld metal, using data from Table 1 and Table 3 as the input data for the simulation of specimen behaviour under tensile loads. When defining plasticity, Abaqus requires the calculation of true stresses and strain based on the values taken from the stress–strain diagram. This approach was used in the numerical models, which will be shown here. Thus, before being used as input data, stress and corresponding strains are converted into their true values using well-known formulas [17]:

(1) $$\sigma_{true}=\sigma \left(1+\varepsilon \right)$$

(2) $$\varepsilon_{true}=ln(1+\varepsilon )$$

where *σ* and *ε* are engineering stress–strain values.

## 3. Finite Element Method Simulations

**Figure 2:**
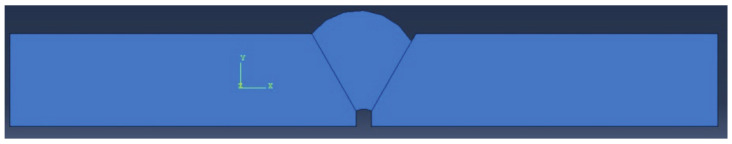
Model with excess weld metal, incomplete root penetration, and undercut.

**Figure 3:**
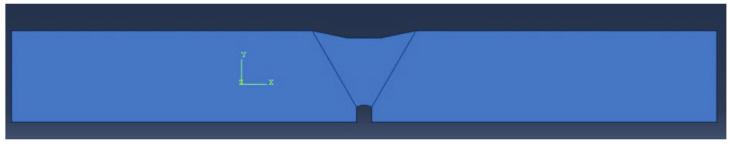
Weld metal sagging and incomplete root penetration.

**Figure 4:**
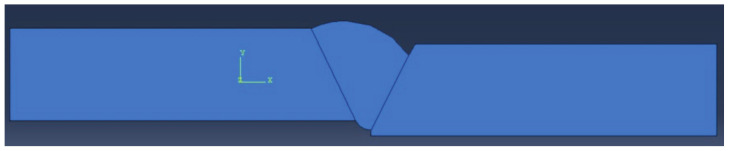
Model with vertical misalignment, incomplete root penetration, and undercut.

**Figure 5:**
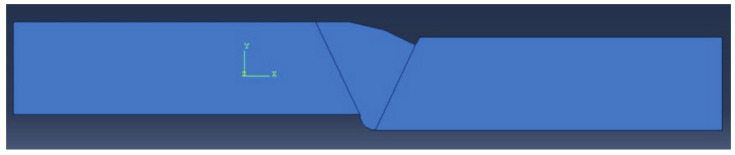
Model with vertical misalignment, undercut, and slight excess root penetration.

In this research, a total of four numerical models were made with combinations of defects, as shown in Figure 2, Figure 3, Figure 4 and Figure 5. The finite elements used were CPS4R, four-node bilinear plane stress quadrilateral elements, and their number was around 7500 to 8900 for all models except for the one in Figure 5, which had 17,800 finite elements.

The defects represented by the model correspond to the ones in real welded plates in terms of both location and dimensions. Regarding the boundary conditions, one vertical edge of each model is fixed. The load is defined on the opposite edge in the form of tension with a magnitude of 100 MPa. Which side of the model is fixed and which is subjected to tensile loads depends on the experiment—it is based on how the specimen is placed in the tensile test machine since not all specimens are facing the same direction. One advantage of the numerical approach is that boundary conditions and loads can easily switch places if there is a need (e.g., when it turns out that the test specimen is placed in the tensile test machine in the opposite direction). In other words, there is always a possibility that the boundary conditions and loads in the model will be defined in one way, but the real specimen will be placed in the tensile test machine in a way that the tensile load is applied on the end of the specimen that was assumed to be fixed in the model and vice versa. Normally, this would not matter, but the geometry resulting from various defects makes the test specimens asymmetric, which means they will behave in different ways depending on which end is fixed and which is subjected to tension.

**Figure 6:**
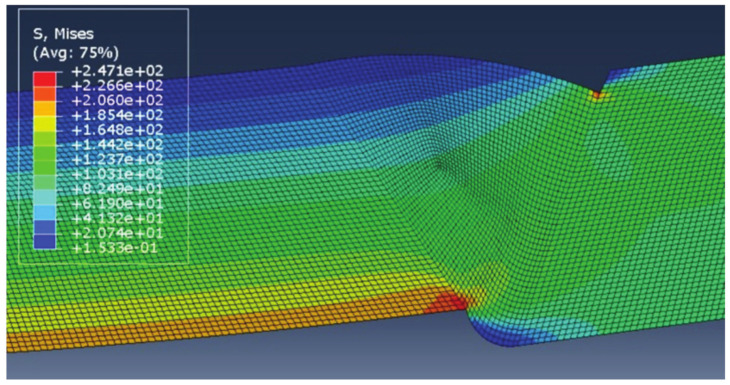
Results for the model with excess weld metal, incomplete root penetration, and undercut.

**Figure 7:**
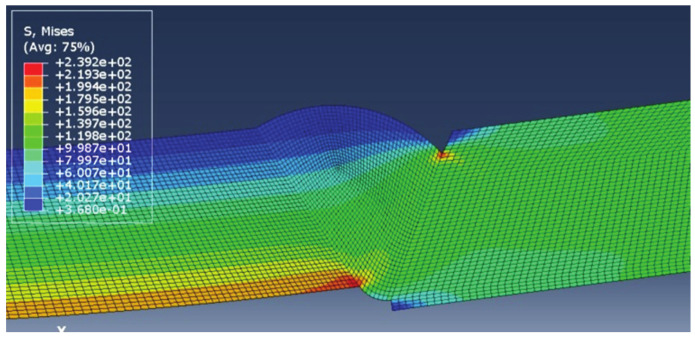
Results for weld metal sagging and incomplete root penetration.

**Figure 8:**
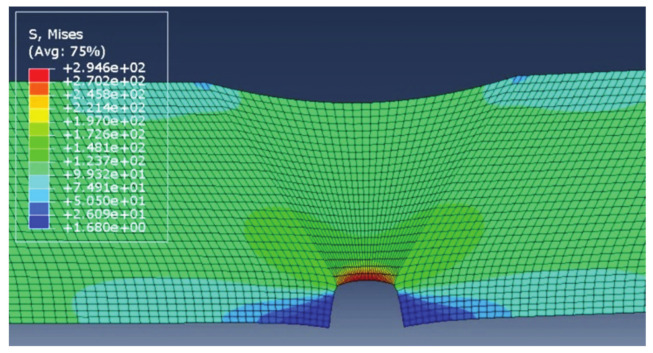
Results for vertical misalignment, incomplete root penetration, and undercut.

**Figure 9:**
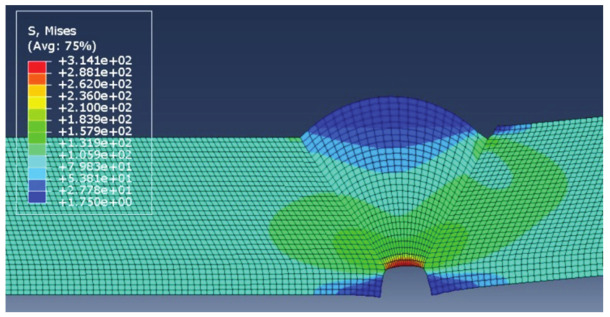
Result for vertical misalignment, undercut, and slight excess root penetration.

The results of each simulation, with the main focus on stress distribution, are shown in Figure 6, Figure 7, Figure 8 and Figure 9. Stress magnitudes are observed in order to determine the locations of stress concentrations and their relation to the defects. These results were also used as a base in the aforementioned previous research [6]. The comparisons made during that stage of the investigation were the inspiration for the following work in order to explain the somewhat unexpected behaviour that was obtained as a result. As can be seen in Figure 6, Figure 7, Figure 8 and Figure 9, stress concentrations are indeed highest at the locations of various defects due to the load direction and irregular geometries. In the case of models without misalignment, stress is concentrated in the weld metal, but is still well below the yield stress; thus no plastic strain is induced. Stresses in the parent material remained in the safe (elastic) region. Regarding the models with misalignments, there are two significantly different observations: they had two different locations with noticeably higher stress, and the highest stresses were observed in the parent material, both slightly exceeding the yield stress, which made these cases less favourable despite the overall lower maximal values compared with the first two models. More particularly interesting results were obtained once tensile tests were performed and numerical and experimental results were compared with each other.

## 4. Experimental Results

**Figure 10:**
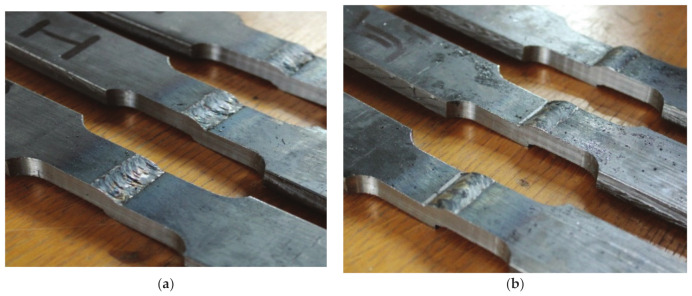

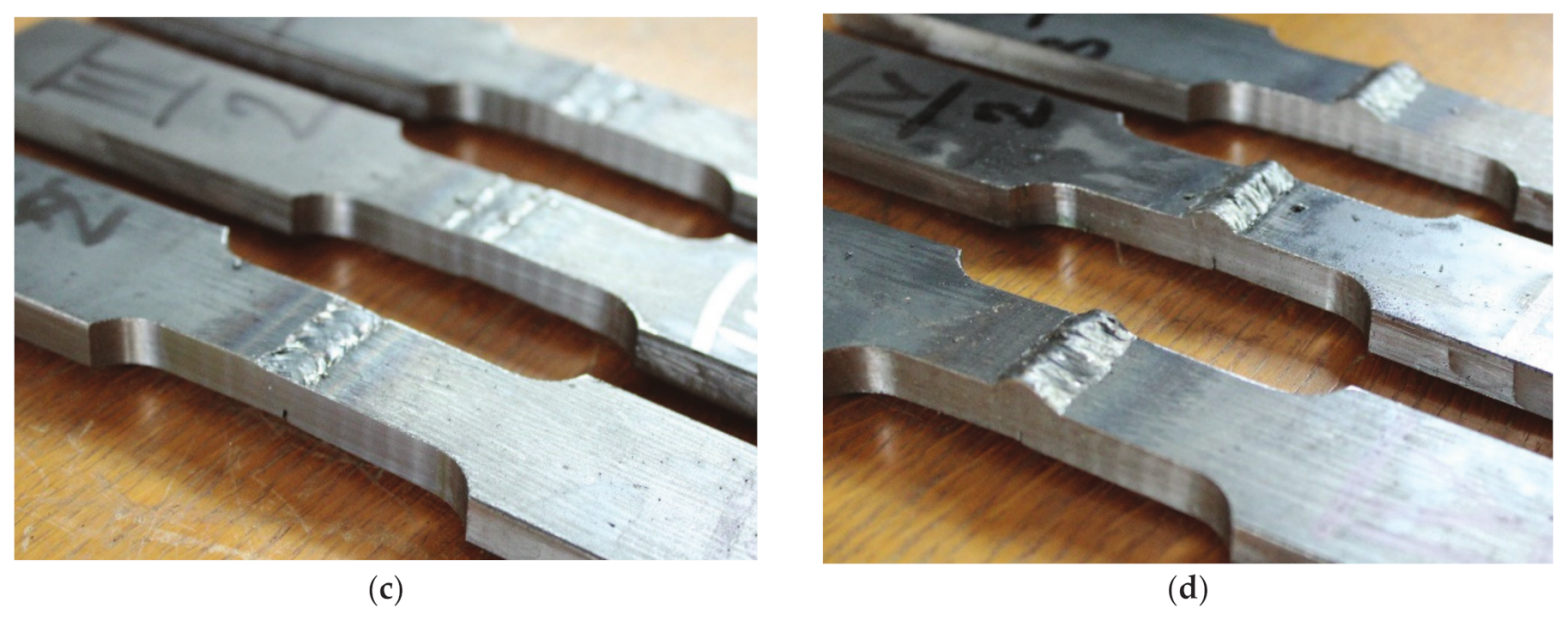
Tensile test specimens with different defect combinations: (**a**) group I—misalignment, undercut, slight excess root penetration; (**b**) group II—misalignment, undercut, incomplete root penetration; (**c**) group III—weld face sagging, incomplete root penetration; (**d**) group IV—excess weld metal, undercut, incomplete root penetration.

It is common practice to verify the results of numerical simulations via experiments, which typically involve numerous types of destructive and nondestructive test methods, as can be seen in [18,19,20,21,22,23,24,25,26,27,28,29]. The experimental stage that followed numerical simulations involved testing four groups of specimens with each group consisting of three specimens with one of four defect combinations. These specimens, with a cross section of 25 × 10 mm, were cut out of plates with corresponding defect combinations (Figure 10a–d).

**Figure 11:**
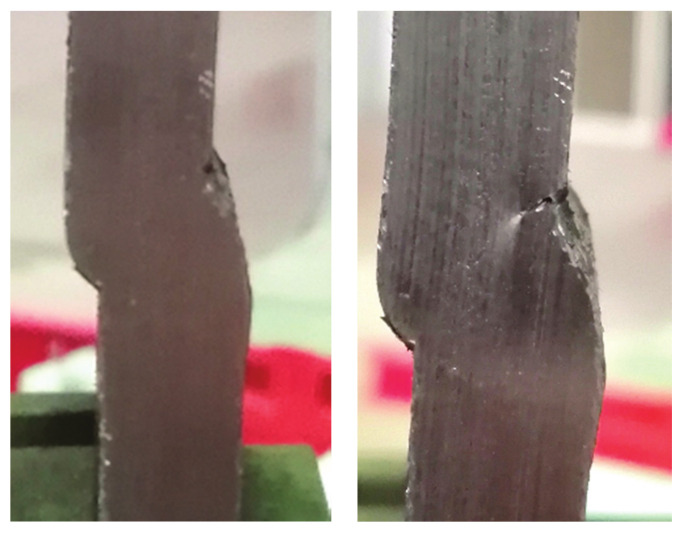
Tensile test specimens during the experiment (misalignment model with slight excess root penetration and undercut) from the initial state to fracture.

**Figure 12:**
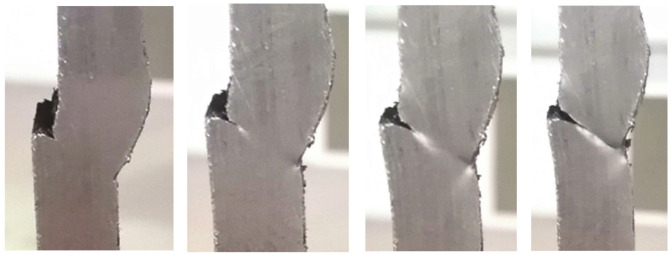
Tensile test specimens during the experiment (misalignment model with incomplete root penetration and undercut) from the initial state to fracture.

**Figure 13:**
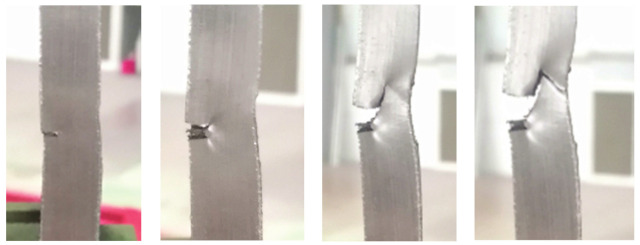
Tensile test specimens during the experiment (weld metal sagging and incomplete root penetration) from the initial state to fracture.

**Figure 14:**
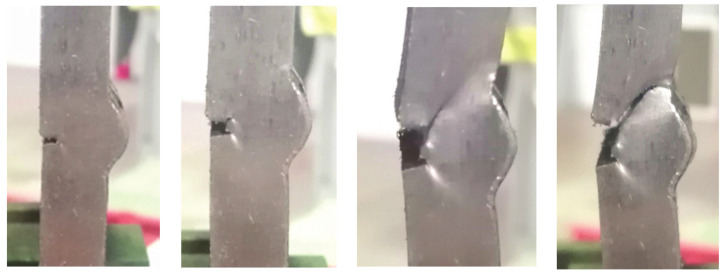
Tensile test specimens during the experiment (excess weld metal, undercut, incomplete root penetration) from the initial state to fracture.

Figure 11, Figure 12, Figure 13 and Figure 14 show the tensile test specimens during different stages of the experiment and illustrate how they deformed until fracture for all four groups. This was used for comparison with the obtained numerical results to determine whether the real stress concentration locations (as well as the location where failure occurred) correspond to the ones obtained by the numerical models.

## 5. Discussion of the Initial Results

Figure 7 and Figure 12 (numerical and experimental results for the misalignment model with incomplete root penetration and undercut), as well as Figure 9 and Figure 14 (numerical and experimental results for the model with excess weld metal, undercut, and incomplete root penetration), indicate only partial agreement between the numerical simulations and the actual experiment. Stress concentrations in both cases are similar and quite high in the undercut (weld face) but different in the root. As can be clearly seen in Figure 12, a crack initiated in the lower part of the root in the tensile test but was on the opposite side of the root (near the ‘higher’ plate) in the numerical model (Figure 7). In both cases, failure would initiate in the root, although at different locations. The way in which the tensile test specimen ultimately failed indicates a significant plastic strain occurring in the undercut after a certain crack length is reached in the root side. The same is implied by the numerical model, wherein stresses in the undercut are only slightly lower than the main stress concentration in the weld root region. Several possible explanations for this phenomenon are considered: the presence of additional internal defects caused by intentionally poor welding; the need for a more detailed information about the heat-affected zone, which is not considered in this research; and the possibility of modelling the same case with an initial crack in the weld root zone, in order to see how this would affect the stress distribution and overall deformation of the model.

Without the misalignment, however, models and experiments have shown very good agreement in terms of deformation. Test specimens have shown crack growth along the fusion line, and the same would have happened in the models if the applied load had been sufficient to cause the parent material to yield (corresponding to the fusion line in this case). This could occur on either side of the specimen since it is the only perfectly symmetrical one. Coincidentally, this did occur during the experiment—one specimen failed via crack initiation and growth on the root side of the fusion line, opposite of the end of the applied force.

Figure 12 illustrates the most interesting result obtained experimentally. Naturally, the numerical model did not indicate this type of failure, and this result presents a unique challenge on its own—explaining how and why the crack suddenly took an almost 90° turn and went from the parent material/heat-affected zone to the much stronger weld metal. The answer will be sought via metallography and fractography tests, which are planned as the next stage of this research.

**Figure 15:**
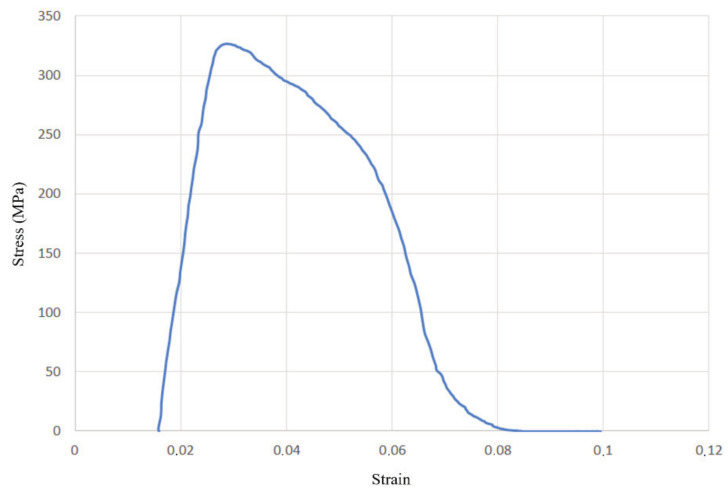
Stress–strain diagram for the group with misalignment, undercut, and excess root penetration (group I).

**Figure 16:**
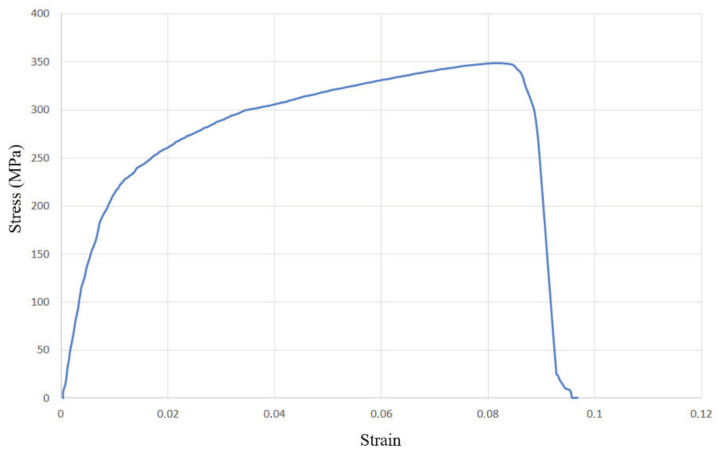
Stress–strain diagram for the group with misalignment, undercut, and incomplete root penetration (group II).

**Figure 17:**
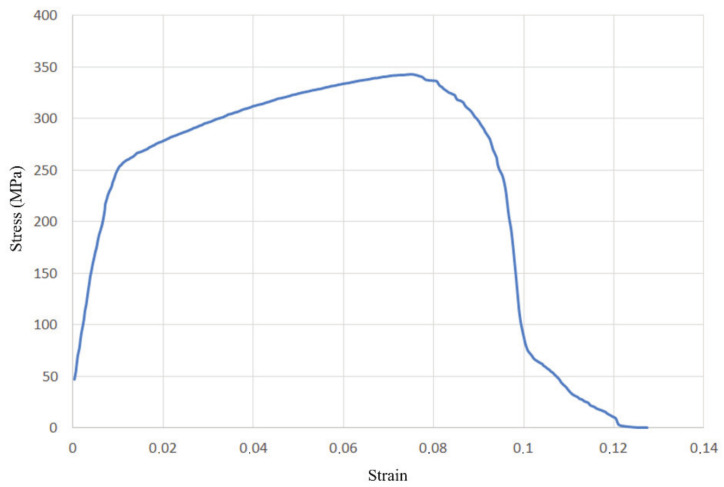
Stress–strain diagram for the group with WM sagging and incomplete root penetration (group III).

Finally, the stress–strain diagrams obtained for each of the four groups are shown in Figure 14, Figure 15, Figure 16 and Figure 17. The first diagram, Figure 15, shows significantly less plasticity (especially taking into account that the whole diagram is ‘displaced’ relative to the origin for reasons related to the force-displacement results; this issue will be properly addressed in the next stages, when higher-quality materials are tested using this methodology). Additionally, the yield stress is very close to the ultimate tensile strength, which in turn is noticeably lower compared with the other three groups. This suggests that welded joints with misalignment and incomplete root penetration are indeed the weakest of the four, either due to the combination of the present defects, or perhaps because of an additional unseen internal defect.

**Figure 18:**
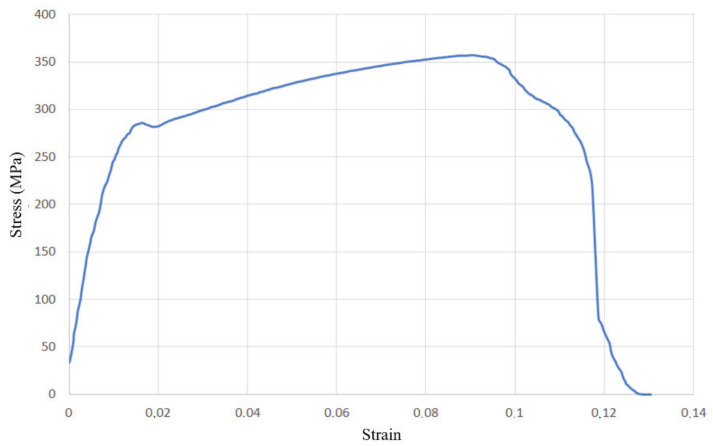
Stress–strain curve for the group with excess WM, undercut, incomplete root penetration (group IV).

**Figure 19:**
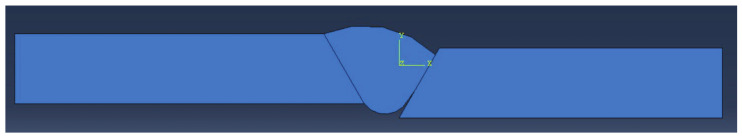
New numerical model geometry, including lack of fusion on the lower side of the welded joint.

From the other three diagrams, Figure 15, Figure 16 and Figure 17, it can be concluded that the test specimens showed very similar behaviour, with nearly identical ultimate tensile strengths and considerable plasticity. The diagram in Figure 18 shows the most prominent yielding, with an easily distinguishable difference between the elastic and plastic regions. The diagrams in Figure 18 and Figure 19 are the most similar, having the same strain of around 9% and with a less obvious yield stress limit. The aforementioned diagram in Figure 17 has a somewhat higher strain, almost 12%. Another interesting aspect related to Figure 18 and Figure 19 is that they represent specimens from two very different groups—with and without misalignment.

## 6. New Version of the Group 2 Specimen Model

**Figure 20:**
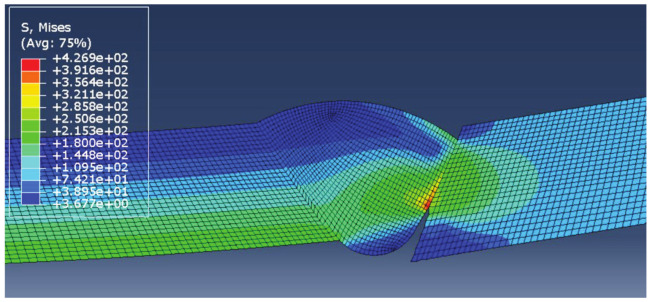

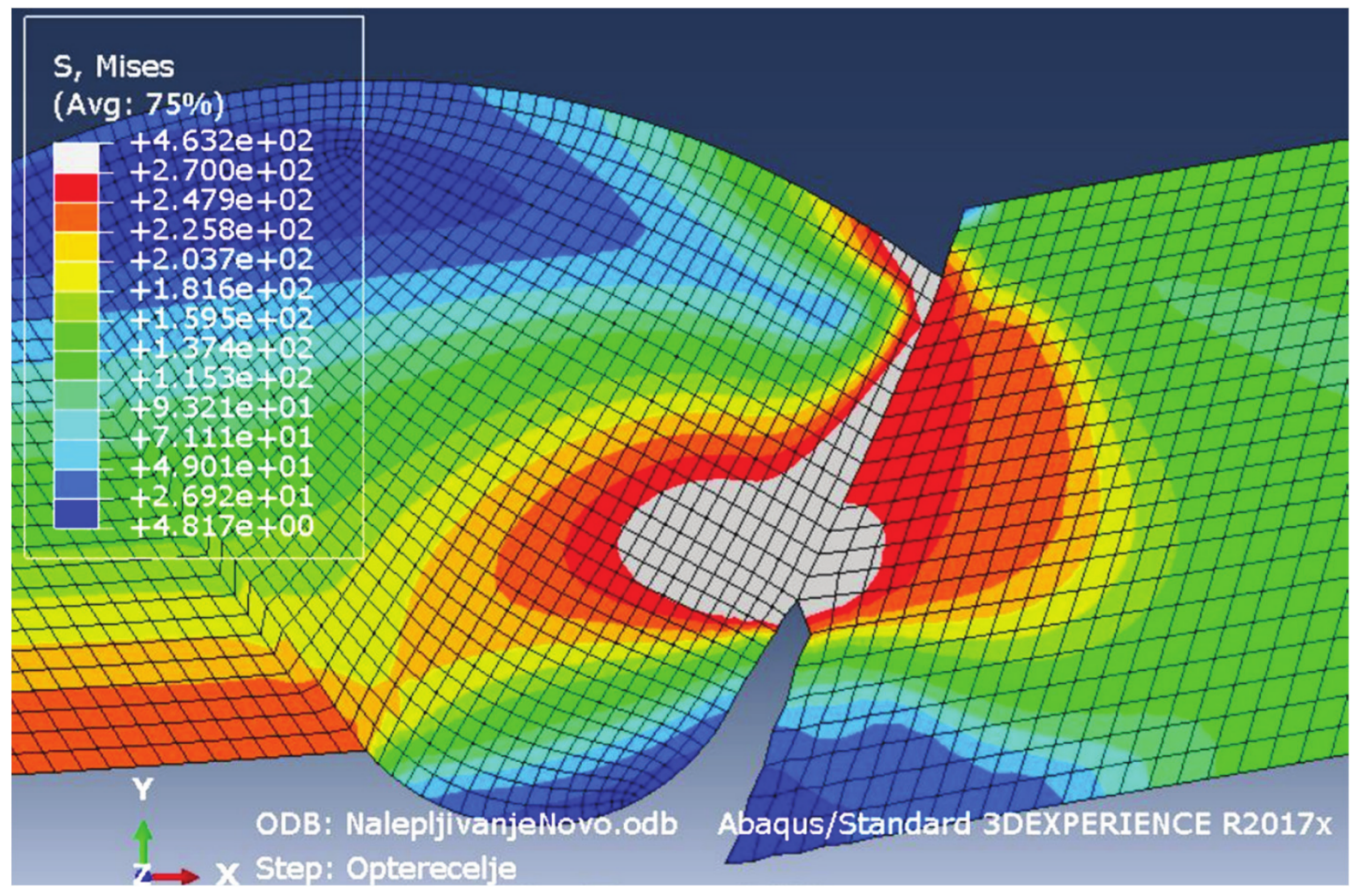
Stress distribution with a tensile load of 100 MPa (**top**) and 120 MPa (**bottom**).

Once the experimental approach confirms that there are noticeable differences in the numerical model and the real specimen, the next step is to include this newly obtained information into the simulation. For this purpose, the depth of lack of fusion is measured and determined to be 4 mm (average value through specimen width). This lack of fusion is then introduced into the numerical model, as shown in Figure 20. It should be noted that this model is the same as the one previously presented in terms of mechanical properties and boundary conditions, and the only changes to its geometry are the added lack of fusion and an increase in gap width, with the second change not having any significant influence on the obtained results.

The results obtained for this new version of the model are shown in Figure 20. It can be clearly seen that there are considerable differences compared with the previous model in terms of both stress concentration locations and stress magnitude. The reason the maximum stress in this case is much higher is that the yield stress of the parent material was exceeded significantly, with stresses reaching as much as 270 MPa. This caused additional deformation in the weld metal, with stresses reaching 427 MPa, very close to filler material yield stress (unlike in the first case, where these values are much lower due to stress concentrators not present in the modified version).

At first glance, the second stress concentration located in the undercut seems negligible, and stresses at this location are insufficient to cause plastic strain. However, as can be seen in the bottom image in Figure 20, the situation changes once the load increases. These results are obtained after the tensile load increases to 120 MPa in order to determine the behaviour of the model under a higher plastic strain. At this point, the regions around the undercut also show stress values above the yield stress of the parent material. It should be noted that some results appear grey since the limit for the maximum stress shown in the image is lowered to 270 MPa. This is done in order to obtain a more detailed stress distribution in the PM region since the stresses in the overmatched weld metal are much higher. At this point, the maximum stresses in the WM reached 463 MPa, slightly above the weld metal yield stress, resulting in plasticity in both regions of the welded joint.

## 7. Conclusions

The aim of this paper is to improve the existing numerical model of a welded joint with a number of surface defects based on real welded joint specimens. The problems with the initial model occur after specimens are experimentally tested, and certain differences are observed between the experimental and numerical results. It is decided to perform additional destructive tests in order to determine whether there are additional factors that contribute to this difference. It is assumed that the difference is influenced by the lack of fusion on the root side of the weld, confirmed by analysing the fractured surface.

- The next stage involves optimisation of the model by introducing newly found defects into the geometry. The obtained results show considerable improvement since the numerical model now behaves in a nearly identical manner as the real specimens in the experiment.
- Stress concentration locations are the same in the experiment and in the new and improved model, and the model itself deforms in a way closely resembling real specimens—with the crack initiating in the weld root—whereas the undercut is subjected to a significant plastic strain, which becomes more prominent with load increase.
- After reaching plasticity in both weld metal and parent material, the behaviour of the numerical model and the experiment show even better mutual agreement.
- In addition to the improved numerical model, based on information obtained on the behaviour of welded joint specimens following the experiment, this model also provides insight into how exactly various defects affect welded joint integrity.
- Incomplete root penetration, combined with lack of fusion, results in a significant adverse effect, whereas the presence of excessive weld metal is completely irrelevant. The undercut is also not of great importance initially, being subjected to compressive stresses due to the combination of load direction and geometry. These stresses only start affecting the welded joint after a significant plastic strain, but at this point, it is already ‘too late’ for the sake of integrity since the welded joint load-bearing cross section has already significantly decreased, causing failure.

This research results in a methodology that can be used in analysing and solving problems previously not considered for welding defects. With a proper combination of experimental and numerical methods, it is possible to accurately describe the behaviour of a welded joint in the presence of several different defects. Future research, in the aforementioned doctoral thesis, will include the application of this method to welded joints of different materials in order to determine how various defect combinations affect integrity since different types of steel can have rather various mechanical properties and resistance to crack growth and so forth.

For this reason, later experiments that will follow this research will also:

- Consider the mechanical properties of the heat-affected zone of the welded joint, in addition to the already-included properties of the parent material and weld metal, which were both used as input parameters for the performed numerical simulations.
- Focus on a more detailed numerical simulation of the heat-affected zone in welded joints. This further emphasises the importance of the experimental part of the research, which will be taken into account in future research, which will also include determining the heat-affected zone mechanical properties via Vickers hardness tests.

## Figures and Tables

**Table 1:** Chemical composition of S235JR steel [6].

Element	C	Mn	P	S	N	Cu
(%)	0.17	1.4	0.035	0.035	0.12	0.55

**Table 2:** Mechanical properties of S235JR steel [6].

Material	Yield Stress *R_eH_* (MPa)	Tensile Strength *R_m_* (MPa)	Thickness (mm)
S235JR	235	360–510	12

**Table 3:** Mechanical properties of VAC 60 [7].

Material	Yield Stress *R_eH_* (MPa)	Tensile Strength *R_m_* (MPa)	Elongation (%)	Toughnessat −40 °C (J)
VAC 60	>410	510–590	>22	>47

**Table 4:** Chemical composition of VAC 60 [7].

Element	C	Si	Mn	P	S
(%)	0.08	0.9	1.5	<0.025	<0.025

**Table 5:** Welding parameters for joints with misalignment.

Layer	Interpass Temperature	Current (A)	Voltage (V)	Welding Speed (mm/s)	Heat Input (kJ/mm)
Root	Below 150	91	18.8	1.7	0.91
Fill 1	Below 150	110	19.4	2.6	0.74
Fill 2	Below 150	120	19.8	1.9	1.12

**Table 6:** Welding parameters for joints without misalignment.

Layer	Interpass Temperature	Current (A)	Voltage (V)	Welding Speed (mm/s)	Heat Input (kJ/mm)
Root	Below 150	111	19.3	2.2	0.87
Fill 1	Below 150	141	23.9	3.2	0.95
Fill 2	Below 150	150	22	4.1	0.71
